# Age-specific survival after breast conservation versus mastectomy for ductal carcinoma in situ of the breast

**DOI:** 10.1007/s10549-026-08097-y

**Published:** 2026-10-20

**Authors:** Jana de Boniface, Robert Szulkin, Per Karlsson, Anna L. V. Johansson

**Affiliations:** 1 Department of Medical Epidemiology and Biostatistics, Karolinska Institutet, Stockholm, SE- 17177 Sweden; 2 Department of Surgery, Capio St. Göran’s Hospital, Stockholm, Sweden; 3 Department of Oncology, Institute of Clinical Sciences, Sahlgrenska Academy, University of Gothenburg, Gothenburg, Sweden; 4 Cancer Registry of Norway, Norwegian Institute of Public Health, Oslo, Norway

**Keywords:** Breast cancer, Ductal carcinoma in situ, Breast conservation, Older age, Survival, Recurrence

## Abstract

**Purpose:**

Breast conservation for invasive breast cancer is associated with better survival and fewer complications than mastectomy, which is especially relevant for older patients. It is unclear whether this can be extrapolated to ductal carcinoma in situ (DCIS).

**Methods:**

This population-based cohort study used data from the Breast Cancer Database Sweden (BCBaSe 3.0) to compare overall survival (OS), breast cancer–specific survival (BCSS) and local recurrence (LR) probabilities in three different age groups (< 60, 60–69, 70 + years) of women with primary DCIS undergoing breast-conserving surgery with radiotherapy (BCS + RT), mastectomy without radiotherapy (Mx-RT), or breast-conserving surgery without radiotherapy (BCS-RT) during 2008–2019. Complete cases (CC) and multiple imputation (MI) analyses were applied.

**Results:**

In 7165 women, treatment was BCS + RT (53.3%), BCS-RT (13.4%), or Mx-RT (33.2%). Estimated 10-year OS was 90.4% (95% CI 89.3–91.5) and BCSS 98.8% (98.3–99.2). After adjustments for patient and tumour factors, including socioeconomic status and comorbidity, Mx-RT was associated with a higher overall mortality than BCS + RT in CC (HR 2.10; 95% CI 1.20–3.67) but not in MI analyses (HR 1.29; 0.95–1.76). In age 70 + years, however, the overall mortality was consistently increased (CC: HR 2.48; 1.09–5.62, MI: HR 1.89; 1.23–2.90). Also BCS-RT was associated with a higher overall mortality than BCS + RT (CC: HR 2.68; 1.34–5.34, and MI: HR 1.72; 1.23–2.39). The 10-year LR probability was low (7.2%, 95% CI 6.3-8.0). Compared to BCS + RT, Mx-RT was associated with a lower (HR 0.18, 95% CI 0.11–0.28) and BCS-RT with a higher LR risk (HR 1.79; 1.31–2.46), especially among ages 70+.

**Conclusion:**

BCS + RT in DCIS appears safe. Acknowledging potential selection bias, BCS + RT may be associated with favourable overall survival outcomes in older women despite a higher risk of local recurrence.

**Supplementary Information:**

The online version contains supplementary material available at https://doi.org/10.1007/s10549-026-08097-y.

## Introduction

In ductal carcinoma in situ (DCIS) of the breast, malignant cells are contained within the basement membrane [1]. Locoregional treatment consists of breast-conserving surgery (BCS) or mastectomy (Mx) with or without breast radiotherapy (RT). DCIS poses specific challenges to BCS due to discontinuous growth and difficult visualization by preoperative imaging. Still, BCS offers significant advantages over Mx, such as a better health-related quality of life, body image, sexual and psychosocial well-being, lower complication rates, and shorter admission and recovery times, and is therefore generally accepted as the recommended surgical option if technically and oncologically feasible [2–7].

In DCIS, local recurrence rates are higher after BCS than after Mx [8, 9]. RT after BCS reduces the absolute 10-year risk of local recurrence by 15.2%, from 28.1% to 12.9%, but does not affect survival rates [10]. The randomized NRG Oncology/RTOG 9804 trial evaluated the omission of RT after BCS for low-risk DCIS and showed a significant reduction of 15-year risk of local recurrence from 15.1% to 7.1% [11]. In Swedish guidelines, patients meeting RTOG low-risk criteria may be considered for omission of RT after BCS [12].

In invasive breast cancer, multiple studies have shown superior overall survival after BCS with RT than after Mx with or without RT [4, 13–15]. This is especially relevant in women aged 70 + years receiving mastectomy, since they have a higher risk of major medical complications which are associated with worse overall survival [16]. It is unclear whether survival outcomes also differ per age group and locoregional treatment for non-invasive breast cancer such as DCIS, after thorough adjustments for potential confounders such as socioeconomic status and comorbidity. This population-based study therefore investigated age-specific survival outcomes and local recurrence risk after BCS with RT compared to Mx without RT and BCS without RT in women operated for DCIS in 2008–2019.

## Methods

We included all women with a primary postoperative DCIS diagnosis registered in the Swedish National Quality Register for Breast Cancer (NKBC) 2008–2019. NKBC records primary non-invasive and invasive breast cancer (International Classification of Diseases, ICD, version-10: C50 and D05) in women residing in Sweden since 2008, including detailed patient, disease, and treatment information. We further utilized the Breast Cancer Database Sweden (BCBaSe 3.0) which is a register linkage between NKBC and the Swedish Cause of Death Register, National Patient Register and Total Population Register. Information on socioeconomic parameters was individually linked from the Longitudinal Integrated Database for Health Insurance and Labour Market Studies (LISA) database at Statistics Sweden.

We retrieved 9752 women with non-invasive breast cancer from BCBaSe 3.0 (eFigure 1). Of these, we kept only patients with a DCIS diagnosis, with or without LCIS. We excluded women with invasive contralateral breast cancer within 6 months of DCIS diagnosis, with a previous invasive diagnosis, or with a re-used personal identification number, leaving 8088 women. A contralateral DCIS diagnosis within 6 months of the first diagnosis was defined as synchronous and was counted once. Disease characteristics were used from the higher nuclear grade and larger extent. Furthermore, we excluded women who were node positive, who received chemotherapy, or with unknown surgery type or date.

### Treatments

Surgical treatment was defined by the final intervention. Planned postoperative RT was available as a binary variable without exact targets and doses. Mx patients with missing RT information and no planned systemic treatment were assumed not to have received RT. Locoregional treatment was categorized as BCS with RT (BCS + RT), mastectomy without RT (Mx–RT) or BCS without RT (BCS–RT). Women receiving Mx + RT (*N* = 150) were excluded, as were 467 women with missing RT information, leaving 7165 for the final analysis (Supplementary Fig. 1).

### Disease information

Information on histopathological DCIS extent (in mm), nuclear grade (categorized as 1/2/3) and mode of detection (screening/clinical) was collected from NKBC. During the study period, the nationwide Swedish screening programme for breast cancer included ages 40–74 years. According to Radiology Therapy Oncology Group (RTOG) criteria, patients were categorized as low risk if nuclear grade 1–2, DCIS extent ≤ 25 mm and screening detected, or as high risk if nuclear grade 3, DCIS extent > 25 mm or clinically detected.

### Patient information

Patient age and region of residence were available from NKBC. Socioeconomic and demographic information was obtained from the LISA database and included educational level one year before diagnosis (≤ 9 years, 10–13 years or > 13 years), family income (low, medium, high), marital status (married/single), and country of birth (Sweden/other). Comorbidity diagnoses 7 years prior to DCIS diagnosis were retrieved from in-patient records of the National Patient Register and combined into a score according to the Royal College of Surgeons’ Charlson Comorbidity Index (CCI; 0,1,2 or 3+) [17, 18]. After exclusions, the cohort included 7782 women, who were analysed in three age groups: <60, 60–69, and 70 + years of age.

### Survival and local recurrence

Dates and causes of death were obtained from the Cause of Death Register. Endpoints were death due to any cause (overall mortality) and death due to breast cancer (breast cancer-specific mortality) with underlying cause of death coded as ICD10: C50. Furthermore, deaths with C50 as contributory cause in combination with registered regional and distant metastases in the National Patient Register, ICD: C77-C79 except C77.3 and C77.9, were also considered as breast cancer deaths. Local recurrence of DCIS or invasive breast cancer was defined as either a record in NKBC, a record of Mx in the National Patient Register at more than 6 months after primary BCS without a registration of a contralateral breast cancer (CBC), or a registered surgery of a local recurrence in the National Patient Register. The date of emigration was obtained from the Total Population Register.

### Statistical methods

Descriptive frequencies and distributions are presented for patient, treatment and disease characteristics and compared with ChiSquare and ANOVA F-tests. Follow-up for death was from date of surgery to date of death or censoring at date of first emigration or end of study (31 Dec 2020), whichever came first. Follow-up for local recurrence was from date of surgery until date of local recurrence, or censoring at date of invasive CBC, death, first emigration or end of study (31 Dec 2020). Cumulative risk of overall death and local recurrence were estimated using the Kaplan-Meier method and compared with the log-rank test. The association between treatment group (BCS + RT, Mx-RT, BCS-RT) and rates of overall death and local recurrence were modelled using Cox proportional hazard regression with time since surgery as the underlying timescale, yielding hazard ratios (HRs) with 95% confidence intervals (CI). To first assess the impact of confounding factors, the models were stepwise adjusted for age, year, region, histopathological extent, mode of detection, nuclear grade, educational level, family income, marital status, country of birth, and CCI. Secondly, to investigate the age-specific associations, interaction between locoregional treatment group and age at diagnosis (< 60, 60–69, 70+) was evaluated for rates of overall death and local recurrence. Additionally, in a first sensitivity analysis, we stratified women by CCI 0–1 and CCI 2+. In a second sensitivity analysis, we included additional censoring due to contralateral breast cancer in the follow-up for death.

Due to a significant proportion of missing values of histopathological extent (46.1%) and nuclear grade (18.1%), we performed multiple imputation with chained equations (MICE) for the Cox regression analyses to avoid a loss in efficiency and minimize bias [19, 20]. We first removed women with missing values in variables with < 1% missing data (endocrine therapy, region, family income, marital status, mode of detection and education), leaving 7075 women in the imputation analysis. Of the remaining women, 55.4% had missing data on either histopathological extent or nuclear grade. We generated 60 imputed datasets with 10 iterations each by replacing missing values with simulated values of histopathological extent and nuclear grade from imputation models based on all predictor variables (histopathological extent, nuclear grade, age, year, treatment group, education, region, mode of detection, family income, country of birth, marital status and CCI), auxiliary variables (endocrine therapy) and the outcome variables (event indicator and the Nelson-Aalen cumulative incidence estimator for time to recurrence, breast cancer death or overall death). The distributions of the imputed values were compared visually to observed distributions. Cox regression was performed on each imputed dataset. Pooled parameter estimates were obtained using Rubin’s rules and can be interpreted under the assumption of missing-at-random. For comparison, Cox regression was also performed on complete cases (*N* = 3157), and the two cohorts from the multiple imputation (MI) and complete case (CC) analyses were described (Supplementary Table 1).

The significance level was 5% and all tests were two-sided. Analyses used R version 4.2.2. Imputation was performed using the R package MICE.

## Results

Out of 9752 women identified from the NKBC, 7165 women fulfilled all eligibility criteria (eFigure 1), 1387 of whom (19.4%) were aged 70 + years. Locoregional treatment consisted of BCS + RT in 3821 (53.3%), BCS-RT in 963 (13.4%), and Mx-RT in 2381 (33.2%) women (Table 1). Among patients aged 70 + years, most received BCS-RT, followed by Mx-RT and BCS + RT. Screening detection was more common in women aged < 60 (76.9%) and 60–69 (84.3%) than those aged ≥ 70 (63.5%). Comorbidity scores of ≥ 2 were more common in women aged ≥ 70 (11.6%) than in those < 60 (4.1%) and 60–69 (6.5%). Median follow-up for death was 71.7 months (0.6-155.9 months).

Women receiving BCS + RT were significantly more often married or in a partnership, had a higher education and a high income, and were diagnosed through screening. While high comorbidity scores were generally rare, the absence of any comorbidity was most common in the BCS + RT group.

**Table 1:** Patient and clinical characteristics of the study population of 7165 women with DCIS in Sweden 2008–2019

	BCS+RT No. (%)	Mx-RT No. (%)	BCS-RT No. (%)	*P*-value
**Total**	3821 (100.0)	2381 (100.0)	963 (100.0)	
**Age at diagnosis**				
< 60	1790 (46.8)	1231 (51.7)	404 (42.0)	< 0.001
60–69	1368 (35.8)	689 (28.9)	296 (30.7)	
70+	663 (17.4)	461 (19.4)	263 (27.3)	
**Calendar year of diagnosis**				
2008–2010	670 (17.5)	552 (23.2)	144 (15.0)	< 0.001
2011–2013	886 (23.2)	588 (24.7)	283 (29.4)	
2014–2016	1023 (26.8)	634 (26.6)	358 (37.2)	
2017–2019	1242 (32.5)	607 (25.5)	178 (18.5)	
**Mode of detection**				
Screening	3223 (84.5)	1631 (68.8)	659 (68.6)	< 0.001
Clinical	589 (15.5)	740 (31.2)	301 (31.4)	
*Missing (% missing)*	*9 (0.2)*	*10 (0.4)*	*3 (0.3)*	
**Histopathological extent**				
Mean, mm (SD)	24.9 (17.4)	51.3 (29.8)	**15.6** (17.1)	< 0.001
Median, mm [Min-Max]	20 [0-149]	48.5 [0-184]	10 [0-120]	
*Missing (% missing)*	*1649 (43.2)*	*1147 (48.2)*	*509 (52.9)*	
**Nuclear grade**				
Grade 1	206 (6.5)	110 (5.6)	221 (31.0)	< 0.001
Grade 2	1268 (39.9)	695 (35.1)	371 (52.1)	
Grade 3	1702 (53.6)	1176 (59.4)	120 (16.9)	
*Missing (% missing)*	*645 (16.9)*	*400 (16.8)*	*251 (26.1)*	
**Region**				
Stockholm-Gotland	927 (24.3)	403 (17.0)	177 (18.5)	< 0.001
Central Sweden	746 (19.6)	511 (21.6)	171 (17.8)	
South-East	398 (10.4)	287 (12.1)	81 (8.5)	
South	641 (16.8)	473 (19.9)	153 (16.0)	
West	712 (18.7)	487 (20.5)	296 (30.9)	
North	389 (10.2)	210 (8.9)	80 (8.4)	
*Missing*	*8 (0.2)*	*10 (0.4)*	*5 (0.5)*	
**Education level**				
≤ 9 years (primary)	574 (15.1)	439 (18.7)	192 (20.1)	< 0.001
10–13 years (secondary)	1627 (42.8)	935 (39.8)	407 (42.7)	
> 13 years (tertiary)	1597 (42.0)	975 (41.5)	354 (37.1)	
*Missing (% missing)*	*23 (0.6)*	*32 (1.3)*	*10 (1.0)*	
**Marital status**				
Married/partner	2228 (58.4)	1303 (55.0)	512 (53.4)	< 0.001
Single	1585 (41.6)	1068 (45.0)	446 (46.6)	
*Missing (% missing)*	*8 (0.2)*	*10 (0.4)*	*5 (0.5)*	
**Country of birth**				
Sweden	3187 (83.4)	1995 (83.8)	797 (82.8)	0.766
Other	634 (16.6)	386 (16.2)	166 (17.2)	
**Family income**				
Low	635 (16.7)	494 (20.8)	239 (24.9)	< 0.001
Middle	1951 (51.2)	1129 (47.6)	462 (48.2)	
High	1227 (32.2)	748 (31.5)	257 (26.8)	
*Missing (% missing)*	*8 (0.2)*	*10 (0.4)*	*5 (0.5)*	
**Charlson comorbidityscore (CCI)** ^**a**^				
CCI 0	3412 (89.3)	2080 (87.4)	823 (85.5)	< 0.001
CCI 1	212 (5.5)	123 (5.2)	61 (6.3)	
CCI 2	161 (4.2)	132 (5.5)	58 (6.0)	
CCI 3+	36 (0.9)	46 (1.9)	21 (2.2)	

^a^ CCI includes diagnoses of comorbidities within 7 years prior to diagnosis

### Overall and breast cancer-specific survival

Overall, 383 deaths were observed, 40 (10.4%) of which were due to breast cancer. The most common causes of death were other malignancies (*N* = 95, 24.8%) and cardiovascular disease (*N* = 78, 20.4%). In 84 cases (21.9%), the primary cause of death was unknown. The estimated 10-year overall survival probability was 90.4% (95% CI 89.3–91.5) for the whole study population: 93.1% (95% CI 91.7–94.5) for the BCS + RT group, 88.0% (95% CI 86.1–90.0) for the Mx-RT and 86.3% (95% CI 82.9–89.8) for the BCS-RT group (log rank *p* < 0.0001).

The estimated 10-year breast cancer-specific survival probability was 98.8% (95% CI 98.3–99.2) for the whole study population: 98.8% (95% CI 98.2–99.4) for the BCS + RT group, 98.5% (95% CI 97.8–99.3) for the Mx-RT and 99.3% (95% CI 98.3–100%) for the BCS-RT group (log rank *p* = 0.25). There were too few events to allow further analysis in adjusted regression models.

### Stepwise adjusted associations with overall survival

In regression analyses including all age groups and based on MI, both Mx-RT and BCS-RT were significantly associated with a higher overall mortality compared to BCS + RT, which remained after stepwise adjustments for patient and disease characteristics and socioeconomic factors (Table 2, models 1–3). After additional adjustment for comorbidity, BCS-RT but not Mx-RT was still associated with worse overall mortality (Table 2, model 4). In the CC analysis, however, the associations for Mx-RT and BCS-RT with worse overall mortality were stronger compared to MI analysis and remained significant in the fully adjusted model. The CC cohort was selected towards later diagnosis years with shorter follow-up and markedly fewer events (Supplementary Table 1). In a sensitivity analysis restricted to women with a CCI of 0–1, thus removing 454 women with CCI ≥ 2, the estimates for overall death remained largely unchanged (Supplementary Table 2). In another sensitivity analysis adding censoring due to CBC, associations between locoregional treatment and overall survival were somewhat stronger and remained significant for Mx-RT also after adjustment for comorbidity (Supplementary Table 3, MI).

**Table 2:** Association between locoregional treatment groups and risk of overall (OS) and local recurrence (LR) with stepwise adjusting for confounders based on the multiple imputation and the complete case analysis, respectively

	Patients (events) No. (No.)	Model 1^a^	Model 2^b^	Model 3^c^	Model 4^d^
		HR (95% CI)	HR (95% CI)	HR (95% CI)	HR (95% CI)
**Multiple Imputation analysis** ^e^					
**Overall death**					
BCS + RT	3787 (130)	1.00 (ref)	1.00 (ref)	1.00 (ref)	1.00 (ref)
Mx-RT	2338 (168)	1.91 (1.51–2.41)	1.44 (1.06–1.96)	1.40 (1.03–1.90)	1.29 (0.95–1.76)
BCS-RT	950 (75)	1.85 (1.38–2.48)	1.81 (1.30–2.51)	1.81 (1.30–2.52)	1.72 (1.23–2.39)
**Local recurrence**					
BCS + RT	3787 (199)	1.00 (ref)	1.00 (ref)	1.00 (ref)	1.00 (ref)
Mx-RT	2338 (31)	0.23 (0.15–0.33)	0.18 (0.11–0.28)	0.18 (0.11–0.28)	0.18 (0.11–0.28)
BCS-RT	950 (83)	1.75 (1.34–2.28)	1.80 (1.32–2.47)	1.79 (1.31–2.46)	1.79 (1.31–2.46)
**Complete case analysis** ^**f**^					
**Overall death**					
BCS + RT	1798 (29)	1.00 (ref)	1.00 (ref)	1.00 (ref)	1.00 (ref)
Mx-RT	1007 (46)	2.46 (1.54–3.94)	2.29 (1.31–3.99)	2.21 (1.27–3.85)	2.10 (1.20–3.67)
BCS-RT	352 (18)	2.87 (1.61–5.13)	3.33 (1.70–6.49)	3.00 (1.53–5.89)	2.68 (1.34–5.34)
**Local recurrence**					
BCS + RT	1798 (58)	1.00 (ref)	1.00 (ref)	1.00 (ref)	1.00 (ref)
Mx-RT	1007 (9)	0.23 (0.12–0.47)	0.16 (0.07–0.37)	0.16 (0.07–0.36)	0.16 (0.07–0.36)
BCS-RT	352 (14)	1.25 (0.68–2.31)	1.27 (0.64–2.53)	1.28 (0.64–2.56)	1.25 (0.62–2.50)

^a^ Model 1 adjusted for age, calendar year of surgery and region of residence

^b^ Model 2 adjusted for same variables as model 1 and histopathological extent, mode of detection and nuclear grade

^c^ Model 3 adjusted for same variables as model 2 and educational level, family income, marital status and country of birth

^d^ Model 4 adjusted for same variables as model 3 and Charlson comorbidity index

^e^ Based on N=7075 women for overall death and local recurrence with imputation of histopathological extent and nuclear grade

^f^ Based on N=3157 women for overall death and local recurrence excluding missing on histopathological extent and nuclear grade

### Age-specific associations with overall survival

**Fig. 1:**
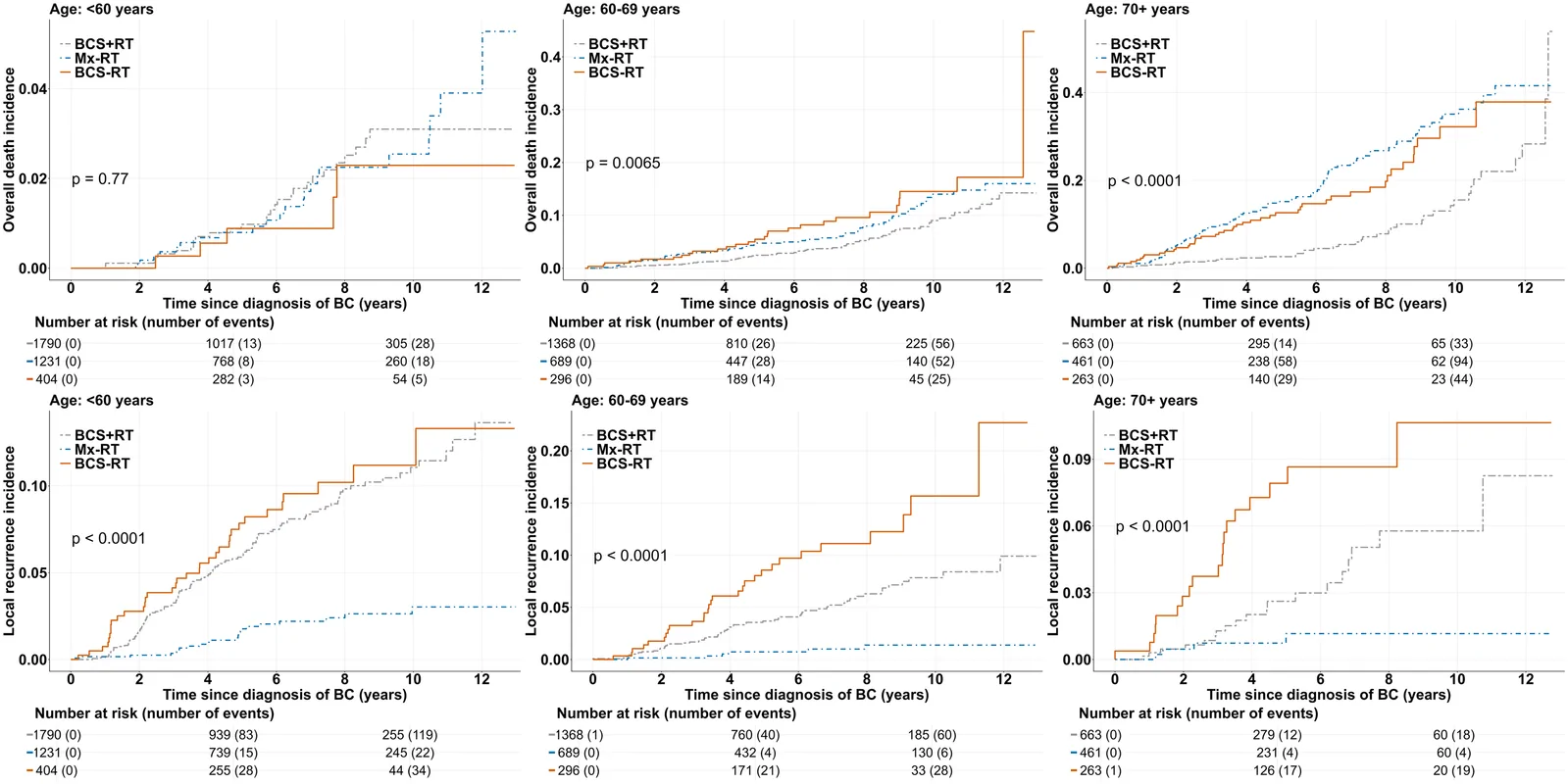
Cumulative risk of overall death and local recurrence by locoregional treatment groups and age

For women aged < 60 or 60–69 years, there was no differences in cumulative risk of death by the three treatment groups (Fig. 1). However, in women aged 70 + years, the cumulative risk of death was significantly higher after Mx-RT and BCS-RT than after BCS + RT. This was corroborated in adjusted regression models, where the strongest significant association between the type of locoregional treatment and overall mortality was observed in women aged 70 + years (Table 3). For Mx-RT, the association among 70 + was present in both analyses (CC: HR 2.48; 1.09–5.62, MI: HR 1.89; 1.23–2.90), while for BCS-RT only the MI analysis was significant (CC: HR 2.14, 0.79–5.82, MI: HR 2.32, 1.47–3.68). For BCS-RT, there was however also a significant association in ages 60–69 years (CC: 3.55, 1.40–9.04; MI: 1.73, 1.06–2.84). In a sensitivity analysis restricted to women with a high (secondary/tertiary) education and screen-detected DCIS up to 30 mm in size, directions of effects and significances were similar, suggesting that any residual confounding is not majorly influencing the main analysis (Supplementary Table 4).

**Table 3:** Association between locoregional treatment groups and risk of overall death and local recurrence by age at diagnosis based on the multiple imputation analysis and the complete case analysis

	Age < 60	Age 60–69	Age 70+
	HR^a^ (95% CI)	HR^a^ (95% CI)	HR^a^ (95% CI)
**Multiple Imputation analysis** ^b^			
**Overall death**			
BCS + RT	1.00 (ref)	1.00 (ref)	1.00 (ref)
Mx-RT	0.77 (0.42–1.40)	1.10 (0.72–1.69)	1.89 (1.23–2.90)
BCS-RT	0.75 (0.28–1.98)	1.73 (1.06–2.84)	2.32 (1.47–3.68)
**Local recurrence**			
BCS + RT	1.00 (ref)	1.00 (ref)	1.00 (ref)
Mx-RT	0.19 (0.11–0.32)	0.12 (0.05–0.30)	0.21 (0.07–0.65)
BCS-RT	1.31 (0.86-2.00)	2.46 (1.51-4.00)	2.58 (1.31–5.05)
**Complete case analysis** ^**c**^			
**Overall death**			
BCS + RT	1.00 (ref)	1.00 (ref)	1.00 (ref)
Mx-RT	1.33 (0.35–4.98)	1.61 (0.76–3.41)	2.48 (1.09–5.62)
BCS-RT	1.34 (0.13–13.71)	3.55 (1.40–9.04)	2.14 (0.79–5.82)
**Local recurrence**			
BCS + RT	1.00 (ref)	1.00 (ref)	1.00 (ref)
Mx-RT	0.17 (0.06–0.47)	N/A	0.74 (0.17–3.25)
BCS-RT	1.06 (0.40–2.76)	0.62 (0.14–2.80)	4.62 (1.17–18.27)

^a^ Model adjusted for age, calendar year of surgery, region of residence, histopathological extent, mode of detection, nuclear grade, educational level, family income, marital status, country of birth and Charlson comorbidity index

^b^ Based on N=7075 women for overall death and local recurrence with imputation of histopathological extent and nuclear grade

^c^ Based on N=3157 women for overall death and local recurrence excluding missing on histopathological extent and nuclear grade

N/A: Not Available as there were no events in this category

### Local recurrence

Overall, 318 local recurrences were registered within 10 years, 204 after BCS + RT, 32 after Mx-RT, and 83 after BCS-RT. The estimated 10-year local recurrence-free survival probability was 92.8% (95% CI 92.0-93.7) for all age groups: 90.9% (95% CI 89.5–92.3) for the BCS + RT group, 97.8% (95% CI 96.9–98.6) for the Mx-RT and 87.4% (95% CI 84.4–90.5) for the BCS-RT group (log rank *p* < 0.0001). Data on the invasiveness of these recurrences were insufficient.

### Stepwise adjusted and age-specific associations with local recurrence

In stepwise adjusted regression models from the MI analysis in all age groups, Mx-RT was independently associated with a lower risk of local recurrence when compared with BCS + RT, while BCS-RT was associated with a higher risk (Table 2). The CC analysis was based on notably fewer events, and showed results similar to the MI analysis, yet the associations were weaker and non-significant for BCS-RT.

The higher local recurrence risks after BCS-RT were most pronounced in women aged 60 years or above and remained only significant in women aged 70 + in the adjusted complete case analysis (Fig. 1; Table 3). In contrast, the association between Mx-RT and a lower risk of local recurrence remained significant only in women below 60 years of age.

### Omission of RT after BCS

The BCS-RT group was explored further regarding low-risk DCIS as defined in the NRG Oncology/RTOG 9804 study. While local recurrence was significantly more common in the high-risk (8 local recurrences in 160 cases) than the low-risk (5 local recurrences in 196 cases) group, this did not affect overall mortality (log rank *p* = 0.23) (Supplementary Fig. 2).

## Discussion

This national population-based study suggests superior overall survival for women with DCIS who receive locoregional treatment by breast-conserving surgery (BCS) followed by radiotherapy (RT) when compared with mastectomy (Mx) without radiotherapy especially in older women. Local recurrence rates were generally low but significantly higher after BCS.

To our knowledge, this is the first report observing superior overall survival rates after BCS + RT in patients with DCIS. The reasons for this could be several. While a role for major postoperative complications was recently suggested, the theoretical presence of dormant cancer cells that could be activated by systemic inflammation caused by complications may be less relevant in the context of non-invasive disease [5]. In the present analysis, postoperative complications were not considered. Another potential reason is unaccounted selection bias affecting surgical decisions. While women receiving BCS + RT had the most favourable socioeconomic characteristics, differences to the Mx-RT were smaller than to the BCS-RT group. Lower socioeconomic status is commonly associated with predictors of worse overall survival such as smoking and obesity, which were unavailable. Similarly, group differences in comorbidity may be more pronounced than reflected by the relatively coarse CCI, potentially contributing to differences in overall survival. However, the fully adjusted model included both socioeconomic factors and CCI, which should at least partly have handled this imbalance between the treatment groups. Only in the imputed data analysis, adjustment for comorbidity weakened the significant association of Mx-RT with overall mortality, which may indicate selection bias or differences in the calendar years of diagnosis.

BCS rates are increasing fuelled both by data showing superior survival rates in patients with invasive breast cancer and by the increasing use of oncoplastic techniques. In the US, the proportion of oncoplastic BCS among all women with DCIS treated by BCS increased from 1.5% in 2005 to 7.6% in 2016 while Mx rates dropped [21]. In Norway, BCS rates in women with DCIS increased from 40% in 1995 to 85% in 2018 [22], which is consistent with our findings. Like the present analysis, the Norwegian study did not present rates of oncoplastic BCS, since specific oncoplastic techniques have only been recorded in registries in more recent years.

Despite a trend towards more BCS in women with DCIS, modern reports on local recurrence rates are rare. Based on Norwegian national data, the 10-year local recurrence rates are 14.3% after standard BCS and 11.2% after complex oncoplastic BCS. Although DCIS extent was higher and high nuclear grade more common in oncoplastic than standard BCS, there was no difference in overall survival (93.0% and 93.3%, respectively) [23]. In general, local recurrence rates after DCIS are declining over time, much in keeping with the corresponding observation in invasive breast cancer but without the hypothesis that improved systemic treatments promote better oncological outcomes. In an analysis from the Netherlands Cancer Registry on 25,719 women with DCIS, the 10-year cumulative incidence of ipsilateral invasive breast cancer decreased from 12.6% to 5.0% in women receiving BCS-RT and from 5.7% to 2.2% in women receiving BCS + RT between 1989 and 1998 and 2009–2021 [24]. Thus, the fact that local recurrence rates were higher after BCS + RT than after Mx-RT in our study may be less relevant in the modern era, since the absolute incidence of local recurrences is low.

Thus far, no subgroup of DCIS has been identified where postoperative RT would not offer improved local control. Selected low-risk patients may discuss omission of RT based on traditional factors such as age, nuclear grade, extent and margins. Ongoing research evaluates genomic tests’ predictive value for RT, such as the Oncotype DX DCIS score or the DCISisionRT^®^ [25–28]. Of note, the present study shows that overall mortality after BCS-RT is inferior to BCS + RT but not to Mx-RT, despite a significantly increased risk of local recurrence.

Several ongoing trials evaluate the oncological safety of omitting surgery in low-risk DCIS (COMET, LORIS, LORD and LORETTA) [29–32]. These efforts at reducing surgical overtreatment for DCIS take the current debate of surgical de-escalation another step forward.

A major strength of this study is its population-based design, which included complete (> 99%) and validated high-quality data from NKBC [33]. The data linkages to other health and socioeconomic registers enabled detailed statistical adjustments for known confounders. We applied multiple imputation to account for missing data on histopathological extent and grade. This helped minimize selection biases and resulted in a nearly complete dataset for the final models, improving both estimation and control for confounding.

This work has some limitations. Some confounders (e.g. smoking, obesity) were not available from national registers; however, since these are commonly associated with socioeconomic status, which was adjusted for, the impact of such factors is likely small. No information on genetic high risk was available, which may motivate more extensive surgery. Women with a genetic predisposition for breast cancer, however, more commonly present with invasive breast cancer than DCIS. Due to the variable selection in national registers, we could neither differentiate between non-invasive and invasive local recurrence, nor between standard and oncoplastic BCS. In addition, the MICE method relies on the missing-at-random (MAR) assumption, which is untestable in the analytical data. As a typical limitation of registry studies, unmeasured factors causing missing not at random cannot be fully excluded. However, our large imputation model including a wide range of variables should increase the likelihood that the imputation step captured the missing mechanism and the correlation structures in the measured variables. The restriction-based sensitivity analysis limited the sample size and should be interpreted with caution due to limited statistical power. Finally, information on margin status is absent in current Swedish registers. While positive or close margins, which should be uncommon since a negative margin of > 2 mm is required by national guidelines, may introduce bias in assessing local recurrence rates, it is unlikely that it would have changed findings regarding survival given that survival rates after BCS (where margin status is most relevant) are higher than after Mx.

In conclusion, BCS + RT in women with a DCIS diagnosis is a locoregional treatment option offering excellent survival outcomes and a low absolute risk of local recurrence, despite a higher relative risk of local recurrence. In older women, BCS + RT may be associated with improved survival outcomes. Thus, the trend towards higher BCS rates in patients with DCIS appears safe.

## Supplementary Information

Below is the link to the electronic supplementary material.

Supplementary Material 1

## Data Availability

No datasets were generated during the current study.
