# Supplementary material for: Age-specific survival after breast conservation versus mastectomy for ductal carcinoma in situ of the breast
Source: Breast Cancer Res Treat. 2026 Oct 20;220(1):10. doi: 10.1007/s10549-026-08097-y (PMC13683482; doi:10.1007/s10549-026-08097-y)
Supplement: Supplementary file 1 [file 10549_2026_8097_MOESM1_ESM.docx]

SUPPLEMENTARY TABLES AND FIGURES

**Supplementary Table 1.** Patient and clinical characteristics of imputed and complete case cohorts.

**Supplementary Table 2.** Sensitivity analysis in patients with CCI 0-1. Association between locoregional treatment groups and risk of overall death and local recurrence by age at diagnosis based on the multiple imputation and the complete case analysis, respectively.

**Supplementary Table 3.** Association between treatment groups and risk of overall (OS) with stepwise adjusting for confounders with censoring for contralateral breast cancer (based on multiple imputation analysis).

**Supplementary Table 4**. Sensitivity analysis restricted to screen-detected DCIS size ≤30 mm in patients with high (secondary or tertiary) education. Association between locoregional treatment groups and risk of overall death and local recurrence by age at diagnosis based on the multiple imputation and the complete case analysis, respectively.

**Supplementary Figure 1.** Flowchart of included patients with DCIS in Sweden 2008-2019.

**Supplementary Figure 2**. Cumulative risk of local recurrence in patients treated by BCS-RT with low-risk versus high-risk DCIS (RTOG criteria).

**Supplementary Table 1.** Patient and clinical characteristics of imputed and complete case cohorts.

|  | **Total cohort^a^**  **(used in multiple imputation analysis)**  **N=7075** | **Cohort excluding missing on tumor size and grade**  **(used in complete-case analysis)**  **N=3157** |
| --- | --- | --- |
|  | **No. (%)** | **No. (%)** |
| **Outcome: Overall death** |  |  |
| Mean follow-up (months) | 74.5 | 51.8 |
| Median follow-up (months) [min, max] | 71.6 [0.6-155.9] | 45.8 [0.9-155.2] |
| Events, deaths | 373 | 97 |
| **Outcome: Local Recurrence** |  |  |
| Mean follow-up (years) | 71.0 | 50.3 |
| Median follow-up (months) [min, max] | 66.9 [0.0-155.9] | 44.5 [0.7-155.2] |
| Events, LR | 313 | 81 |
| **Surgery type** |  |  |
| BCS+RT | 3787 (53.5) | 1798 (57.0) |
| Mx-RT | 2338 (33.0) | 1007 (31.9) |
| BCS-RT | 950 (13.4) | 352 (11.1) |
| **Histopathological extent/Tumor Size** |  |  |
| Mean, mm (SD) | 32.27 [25.85] | 31.56 [25.21] |
| Median, mm [Min-Max] | 25 [0-184] | 25 [0-180] |
| *Missing (% missing)* | *3251 (46.0%)* | *0 (0.0%)* |
| **Nuclear grade** |  |  |
| Grade 1 | 530 (9.1) | 275 (8.7) |
| Grade 2 | 2303 (39.8) | 1268 (40.2) |
| Grade 3 | 2960 (51.1) | 1614 (51.1) |
| *Missing (% missing)* | *1282 (18.1%)* | *0 (0.0%)* |
| **Mode of detection** |  |  |
| Screening | 5473 (77.4) | 2481 (78.6) |
| Clinical | 1602 (22.6) | 676 (21.4) |
| **Age at diagnosis** |  |  |
| <60 | 3377 (47.7) | 1468 (46.5) |
| 60-69 | 2330 (32.9) | 1020 (32.3) |
| 70+ | 1368 (19.3) | 669 (21.2) |
| **Calendar year of diagnosis** |  |  |
| 2008-2010 | 1300 (18.4) | 196 (6.2) |
| 2011-2013 | 1714 (24.2) | 270 (8.6) |
| 2014-2016 | 1977 (27.9) | 1037 (32.8) |
| 2017-2019 | 2084 (29.5) | 1654 (52.4) |
| **Region** |  |  |
| Stockholm-Gotland | 1492 (21.1) | 639 (20.2) |
| Central Sweden | 1419 (20.1) | 1121 (35.5) |
| South-East | 758 (10.7) | 295 (9.3) |
| South | 1243 (17.6) | 503 (15.9) |
| West | 1486 (21.0) | 305 (9.7) |
| North | 677 (9.6) | 294 (9.3) |
| **Education level** |  |  |
| ≤9 years (primary) | 1200 (17.0) | 505 (16.0) |
| 10-13 years (secondary) | 2962 (41.9) | 1321 (41.8) |
| >13 years (tertiary) | 2913 (41.2) | 1331 (42.2) |
| **Marital status** |  |  |
| Married/partner | 4010 (56.7) | 1781 (56.4) |
| Single | 3065 (43.3) | 1376 (43.6) |
| **Country of birth** |  |  |
| Sweden | 5944 (84.0) | 2641 (83.7) |
| Other | 1131 (16.0) | 516 (16.3) |
| **Family income** |  |  |
| Low | 1335 (18.9) | 569 (18.0) |
| Middle | 3524 (49.8) | 1543 (48.9) |
| High | 2216 (31.3) | 1045 (33.1) |

^a^ Total cohort N=7075 excludes missing values on other variables than tumor size and nuclear grade.

**Supplementary Table 2.** Sensitivity analysis in patients with CCI 0-1. Association between locoregional treatment groups and risk of overall death and local recurrence by age at diagnosis based on the multiple imputation and the complete case analysis, respectively.

|  | **Age <60** | **Age 60-69** | **Age 70+** |
| --- | --- | --- | --- |
|  | **HR^a^ (95% CI)** | **HR^a^ (95% CI)** | **HR^a^ (95% CI)** |
| **Multiple Imputation analysis^b^** |  |  |  |
| **Overall death** |  |  |  |
| BCS+RT | 1.00 (ref) | 1.00 (ref) | 1.00 (ref) |
| Mx-RT | 0.84 (0.44-1.59) | 1.01 (0.63-1.62) | 1.74 (1.09-2.76) |
| BCS-RT | 0.74 (0.25-2.18) | 1.85 (1.07-3.19) | 2.31 (1.40-3.79) |
| **Local recurrence** |  |  |  |
| BCS+RT | 1.00 (ref) | 1.00 (ref) | 1.00 (ref) |
| Mx-RT | 0.19 (0.11-0.32) | 0.12 (0.05-0.32) | 0.18 (0.05-0.62) |
| BCS-RT | 1.26 (0.82-1.95) | 2.64 (1.60-4.37) | 2.39 (1.17-4.87) |
|  |  |  |  |
| **Complete case analysis^c^** |  |  |  |
| **Overall death** |  |  |  |
| BCS+RT | 1.00 (ref) | 1.00 (ref) | 1.00 (ref) |
| Mx-RT | 1.52 (0.33-7.03) | 1.29 (0.57-2.90) | 2.37 (0.96-5.84) |
| BCS-RT | 2.07 (0.19-22.36) | 4.02 (1.32-12.18) | 1.98 (0.67-5.88) |
| **Local recurrence** |  |  |  |
| BCS+RT | 1.00 (ref) | 1.00 (ref) | 1.00 (ref) |
| Mx-RT | 0.14 (0.05-0.44) | N/A | 0.53 (0.09-2.92) |
| BCS-RT | 0.95 (0.33-2.72) | 0.40 (0.05-3.20) | 3.88 (0.92-16.34) |

^a^ Model adjusted for age, calendar year of surgery, region of residence, tumor size, mode of detection, Nottingham grade, educational level, family income, marital status, country of birth and Charlson comorbidity index.

^b^ Based on N=7075 women for overall death and local recurrence with imputation of tumor size and nuclear grade.

^c^ Based on N=3157 women for overall death and local recurrence excluding missing on tumor size and nuclear grade.

N/A: Not Available as there were no events in this category.

**Supplementary Table 3.** Association between treatment groups and risk of overall (OS) with stepwise adjusting for confounders with censoring for contralateral breast cancer (based on imputation analysis).

|  | **Patients**  **(events)**  **No. (No.)** | **Model 1** | **Model 2** | **Model 3** | **Model 4** |
| --- | --- | --- | --- | --- | --- |
|  |  | **HR (95% CI)** | **HR (95% CI)** | **HR (95% CI)** | **HR (95% CI)** |
| **Overall death** |  |  |  |  |  |
| BCS+RT | 3787 (116) | 1.00 (ref) | 1.00 (ref) | 1.00 (ref) | 1.00 (ref) |
| Mx-RT | 2338 (162) | 2.09 (1.64-2.66) | 1.64 (1.20-2.25) | 1.60 (1.17-2.19) | 1.47 (1.07-2.03) |
| BCS-RT | 950 (74) | 2.07 (1.53-2.79) | 1.94 (1.38-2.73) | 1.94 (1.38-2.72) | 1.84 (1.31-2.58) |

Based on N=7075 women for overall death.

1) Model 1 adjusted for age, calendar year of surgery and region of residence.

2) Model 2 adjusted for same variables as 1) and tumor size, mode of detection and Nottingham grade.

3) Model 3 adjusted for same variables as 2) and educational level, family income, marital status and country of birth.

4) Model 4 adjusted for same variables as 3) and Charlson comorbidity index.

**Supplemental Table 4.** Sensitivity analysis restricted to screen-detected DCIS size ≤30 mm in patients with high (secondary or tertiary) education. Association between locoregional treatment groups and risk of overall death and local recurrence by age at diagnosis based on the multiple imputation and the complete case analysis, respectively.

|  | **Age <60** | **Age 60-69** | **Age 70+** |
| --- | --- | --- | --- |
|  | **HR^a^ (95% CI)** | **HR^a^ (95% CI)** | **HR^a^ (95% CI)** |
| **Multiple Imputation analysis^b^** |  |  |  |
| **Overall death** |  |  |  |
| BCS+RT | 1.00 (ref) | 1.00 (ref) | 1.00 (ref) |
| Mx-RT | 0.87 (0.20-3.78) | 1.67 (0.75-3.73) | 2.81 (1.22-6.45) |
| BCS-RT | 0.45 (0.08-2.56) | 2.06 (1.03-4.12) | 2.52 (1.19-5.32) |
| **Local recurrence** |  |  |  |
| BCS+RT | 1.00 (ref) | 1.00 (ref) | 1.00 (ref) |
| Mx-RT | 0.18 (0.05-0.66) | N/A | N/A |
| BCS-RT | 1.34 (0.77-2.32) | 3.32 (1.82-6.05) | 2.42 (0.93-6.27) |
|  |  |  |  |
| **Complete case analysis^c^** |  |  |  |
| **Overall death** |  |  |  |
| BCS+RT | 1.00 (ref) | 1.00 (ref) | 1.00 (ref) |
| Mx-RT | 0.98 (0.09-11.09) | 3.45 (0.88-13.46) | 3.86 (0.80-18.56) |
| BCS-RT | N/A | 2.79 (0.57-13.77) | 2.38 (0.26-21.63) |
| **Local recurrence** |  |  |  |
| BCS+RT | 1.00 (ref) | 1.00 (ref) | 1.00 (ref) |
| Mx-RT | 0.36 (0.05-2.84) | N/A | N/A |
| BCS-RT | 2.53 (0.49-13.09) | 1.22 (0.10-15.21) | 4.79 (0.74-31.20) |

^a^ Model adjusted for age, calendar year of surgery, region of residence, histopathological extent, mode of detection, nuclear grade, educational level, family income, marital status, country of birth and Charlson comorbidity index.

^b^ Based on N=2897 women for overall death and local recurrence with imputation of histopathological extent and nuclear grade.

^c^ Based on N=1305 women for overall death and local recurrence excluding missing on histopathological extent and nuclear grade.

N/A: Not Available as there were no events in this category.

**Supplementary Figure 1.** Flowchart of included and excluded patients.

Non-Invasive BC diagnosis (DCIS) 2008-2019

registered in Swedish National Quality Register for Breast Cancer (NKBC)

N=9752 women

N=8088 women with DCIS

Excluded:

-330 women with invasive contralateral breast cancer within 6 months of DCIS diagnosis

-803 women with an in situ diagnosis other than DCIS within 6 months of DCIS diagnosis

-527 women with previous invasive diagnosis (ipsilateral or contralateral) in the Swedish Cancer Register prior to DCIS

-4 women with a re-used personal identification number

Excluded:

-129 women with positive lymph node status at diagnosis

-86 women with positive lymph nodes on histopathology report

-78 women with registered receipt of chemotherapy

-13 women with unknown surgery type or unknown date of surgery

Final analytical sample:

N=**7165** women with BCS+RT, BCS-RT, Mx-RT

For imputation analysis:

N=**7075**

(excluded 90 women with missing values in variables with <1% missing)

N=7782 women

Excluded:

-467 women with BCS and missing information on radiotherapy

-150 women with Mx+RT

**Supplementary Figure 2.** Cumulative risk of local recurrence in patients treated by BCS-RT with low-risk versus high-risk DCIS (RTOG criteria).
